# Liver Involvement Associated with Dengue Infection in Adults in Vietnam

**DOI:** 10.4269/ajtmh.2010.10-0090

**Published:** 2010-10-05

**Authors:** Dinh The Trung, Le Thi Thu Thao, Tran Tinh Hien, Nguyen The Hung, Nguyen Ngoc Vinh, Pham Tran Dieu Hien, Nguyen Tran Chinh, Cameron Simmons, Bridget Wills

**Affiliations:** Hospital for Tropical Diseases, Ho Chi Minh City, Vietnam; University of Medicine and Pharmacy of Ho Chi Minh City, Vietnam; Oxford University Clinical Research Unit, Hospital for Tropical Diseases, Ho Chi Minh City, Vietnam; Centre for Clinical Vaccinology and Tropical Medicine, Oxford University, Oxford, United Kingdom

## Abstract

Globally, the number of adults hospitalized with dengue has increased markedly in recent years. It has been suggested that hepatic dysfunction is more significant in this group than among children. We describe the spectrum and evolution of disease manifestations among 644 adults with dengue who were prospectively recruited on admission to a major infectious disease hospital in southern Vietnam and compare them with a group of patients with similar illnesses not caused by dengue. Transaminase levels increased in virtually all dengue patients and correlated with other markers of disease severity. However, peak enzyme values usually occurred later than other complications. Clinically severe liver involvement was infrequent and idiosyncratic, but usually resulted in severe bleeding. Chronic co-infection with hepatitis B was associated with modestly but significantly increased levels of alanine aminotransferase, but did not otherwise impact the clinical picture.

## Introduction

Dengue is the most widely distributed mosquito-borne viral infection of humans, affecting up to 100 million persons each year across the tropical world.^1^,^2^ Infection with any of the four dengue viral serotypes may result in asymptomatic infection or may cause a range of disease manifestations from non-specific fever to a syndrome characterized by increased vascular permeability, thrombocytopenia, and deranged hemostasis. In severe cases, the increased vascular permeability results in circulatory compromise and the patient may develop potentially life-threatening dengue shock syndrome (DSS).^2^–^4^ No specific antiviral therapy is available but the physiologic derangements are transient, and most patients recover fully if supported with parenteral fluid therapy during the period of maximal vascular leakage. Current mortality rates for DSS are less than 1% in experienced hands.^5^–^7^

Dengue shock syndrome is an important cause of hospitalization among children living in dengue-endemic areas. However, in general, severe bleeding is not a major problem in this group, despite often profound thrombocytopenia and clear evidence of a coagulopathy.^8^–^10^ In recent years, there has been a notable increase in the number of adults with dengue requiring hospitalization in Asia and South America.^6^,^11^,^12^ Dengue shock syndrome appears to be less frequent in adults than children, possibly reflecting age-dependent differences in intrinsic vascular permeability, but there is anecdotal evidence to suggest that bleeding manifestations and hepatic dysfunction are both more common in older age groups.^6^,^11^,^13^ There have been few prospective studies focused on disease manifestations in adults, and there is little systematic data describing the clinical profile of disease in older patients or those with co-morbidities, or detailing the evolution of clinical and laboratory parameters through the various stages of the infection.

Hepatic dysfunction is a well recognized feature of dengue infections, often demonstrated by hepatomegaly and mild-to-moderate increases in transaminase levels although jaundice and acute liver failure are generally uncommon.^14^–^17^ Biopsy specimens obtained from a small number of patients with DSS who died have shown a variety of patterns including microvesicular steatosis, hepatocellular necrosis with associated councilman bodies, Kupffer cell destruction, and inflammatory infiltrates at the hepatic portal tracts.^18^,^19^ Dengue antigens and viral RNA have been demonstrated in some of these fatal cases, and dengue viruses have been isolated occasionally from hepatic tissue.^19^–^22^ However, biopsy specimens are rarely obtained from less severe cases and the relevance of these findings to the broad spectrum of dengue infections remains uncertain. Debate continues as to whether dengue associated hepatic dysfunction indicates a direct viral effect, arises secondary to an aggressive host immune response to the virus, or reflects a complex interaction of these two mechanisms.

One factor that may influence the pattern of disease seen in adults is the greater likelihood of underlying chronic diseases, potentially compounding the effects of the acute infection. Chronic viral hepatitis is common among adults in many tropical and sub-tropical countries where dengue is endemic, and it has been postulated that dengue infection occurring on a background of chronic infection with hepatitis B virus (HBV) or hepatitis C virus (HCV) may result in more severe liver dysfunction and/or hemorrhage than is usual in non-infected persons. However, the evidence to date is conflicting; two small studies indicated no effect,^14^,^15^ and one study suggested that concomitant HBV infection may result in greater hepatic dysfunction.^23^

We conducted a large prospective study to describe the spectrum of symptomatic dengue disease among adults admitted to a major infectious disease hospital in southern Vietnam. We focused particularly on liver involvement, aiming to assess relationships with vascular leakage and bleeding manifestations. We also examined the question of whether concomitant infection with HBV or HCV influenced the overall severity of the clinical picture.

## Methods

### Patients and clinical methods.

Patients more than 14 years of age admitted to the Hospital for Tropical Diseases in Ho Chi Minh City, Vietnam, with clinically suspected dengue were eligible for enrollment in the study after written informed consent was obtained from a patient or a relative in the case of those with coma or very severe disease. Ethical approval was obtained from the Scientific and Ethical Committee of the Hospital for Tropical Diseases and the Oxford University Tropical Research Ethics Committee, United Kingdom. The hospital admits approximately 5,000 adults with dengue each year. Recruitment was targeted to include all patients with suspected dengue admitted to the Adult Intensive Care Unit (AICU) plus a group of 200 female patients and 200 male patients with suspected dengue admitted to particular infectious disease wards.

Within 24 hours of admission, designated senior doctors on these wards tried to recruit all patients admitted to specific bays with suspected dengue; diagnostic criteria were not specified because we aimed to include the full spectrum of hospitalized cases. Detailed clinical information was collected daily for the duration of the hospital stay by one study physician by using a standard case report form particularly focused on liver involvement, bleeding manifestations, and evidence of vascular leakage. A venous blood sample was obtained each day for clinical and diagnostic investigations as detailed below. Additional investigations and management were at the discretion of the treating physicians. Patients in whom complications developed were transferred from the infectious disease wards to the AICU and continued with daily clinical assessment by the same study physician. After discharge all patients were invited to attend for review approximately one month after the illness onset.

Acute liver failure was diagnosed in patients without evidence of pre-existing cirrhosis in whom any degree of mental alteration with increased liver enzyme levels and a coagulopathy (as demonstrated by an international normalized ratio ≥ 1.5) developed.^24^ Following the new World Health Organization guidelines, vascular leakage was classified primarily according to whether hypovolemic shock developed in the patient.^4^ We defined shock as hypotension or narrowing of the pulse pressure with impaired peripheral perfusion that was thought to be caused by plasma leakage, not bleeding, and required volume resuscitation. We also classified the dengue patients without shock into two categories: those with and without clear evidence of vascular leakage.

Criteria for inclusion in the vascular leakage without shock group were either hemoconcentration ≥ 20% between the peak recorded hematocrit and a baseline value obtained before day 3 of illness or the local population mean for age and sex (reference database for 850 healthy adults in Vietnam, unpublished data), and/or evidence of fluid accumulation on a radiograph or ultrasound image obtained at the time of defervescence. Patients without hemoconcentration who had more than three days of serial hematocrit data and a properly timed negative radiograph/ultrasound image were classified as having dengue without evidence of vascular leakage, and patients with insufficient data were considered unclassifiable. The severity of bleeding manifestations was also coded retrospectively into one of the following four categories: no clinical bleeding detected, minor skin bleeding only (petechiae or bruising at venipuncture sites), minor mucosal bleeding with or without minor skin bleeding, and severe bleeding defined as any bleeding requiring an intervention or any bleeding into vital organs (e.g., intracranial bleeding).

### Laboratory methods.

A complete blood count was obtained at least once a day in the routine hematology laboratory. On AICU hematocrit measurements were also made as frequently as required for clinical management. Albumin, bilirubin, and transaminase levels were determined at intervals during the course of disease: the early febrile period (days 1–3 of illness), the critical period for complications such as shock (days 4–6), and the convalescent period (days 7–10). We also performed coagulation screening tests for the first 200 patients at these time points, as well as for any patient with a clinical indication. Plasma was separated and stored at –80°C within six hours of sampling for subsequent analysis in batches in a specialized hematology laboratory. All tests were repeated at the follow-up visit.

Diagnostic tests for HBV and HCV were performed on stored convalescent-phase or follow-up samples by using AxSYM reagents (Abbott, Abbott Park, IL). Antibodies to hepatitis B core protein (HBc) were measured in an initial screen in all patients, and tests for hepatitis B surface antigen and IgM against HBc were performed if the first antibody test result was positive. Antibodies to HCV were measured in samples from the first 200 patients enrolled in the study.

For diagnosis of dengue, IgM- and IgG-capture enzyme-linked immunosorbent assays were performed for paired specimens. A dengue virus polymerase chain reaction and a nonstructural protein 1 enzyme-linked immunosorbent assay were also conducted for the first specimen if obtained within the first five days of illness, or if serologic results were negative or inconclusive. The techniques used and interpretation of results were performed as reported.^25^ Patients in whom all dengue diagnostic results were conclusively negative, in whom there was no evidence of any other infectious disease, and who recovered without antibiotic therapy, were considered to have had other febrile illnesses (OFIs).

### Statistical analysis.

Data for clinical characteristics and laboratory results were compared between different patient groups by using the chi-square test or Fisher's exact test for categorical variables and the Mann-Whitney test or the Cuzick test for trend for continuous variables. Within-patient comparisons of different parameters or of the same parameter measured at different time points were performed by using the Wilcoxon signed-rank test. Spearman's rank correlation was used to evaluate relationships between continuous variables assessed concurrently. All statistical computations were carried out using SPSS version 14 (SPSS, Inc., Chicago, IL) and Stata SE Version 8 (StataCorp., College Station, TX).

## Results

During September 2006–September 2008, 740 persons were recruited into the study, including 340 enrolled in AICU (90% of those eligible) plus 200 male and 200 female patients (approximately 50% of those eligible from the designated bays on the relevant infectious disease wards). Dengue was confirmed in 644 (87%) of 740 patients and 47 patients (6%) were classified as having OFIs. The infecting serotype was identified in 207 (32%) patients: dengue 1 virus in 108 patients, dengue 2 virus in 65 patients, dengue 3 virus in 30 patients, and dengue 4 virus in 4 patients. An alternative diagnosis (e.g., malaria, bacterial sepsis) was confirmed or suspected in 13 patients (2%), all of whom had negative dengue diagnostic results. In the remaining 36 patients (5%), the results of serologic and virologic diagnostic tests were inconclusive.

Among the 644 patients with confirmed dengue, 55 (9%) were admitted first to one of the infectious disease wards but transferred to the AICU during their hospital stay. Six patients with dengue died, one patient without shock died of acute liver failure, and complications including severe bleeding, encephalopathy, renal failure and/or metabolic acidosis developed in five patients with shock; they died despite supportive care. The other patients in the study fully recovered and 296 (40%) of the survivors attended follow-up. The remainder of this report focuses on patients with confirmed dengue or OFIs.

Demographic and summary clinical data are shown in Table 1. Although dengue and patients with OFIs were of similar age, dengue patients with shock were slightly younger than those without shock and presented approximately one day later in the evolution of their illness. Shock did not occur in the OFI group, and there were few bleeding or hepatic manifestations, although 5 of 47 were admitted briefly to the AICU for observation during the critical period for shock. All the 112 dengue patients with shock manifested some evidence of clinical bleeding, including a mucosal site in more than 70% of the patients. However, among the 55 dengue patients who experienced severe bleeding, most (40 patients, 73%) did not have shock.

In this group, one patient had intracranial bleeding confirmed by computed tomography scan, 26 required transfusion of blood products for major mucosal bleeding (19 erythrocyte transfusions and 7 fresh-frozen plasma and/or platelet transfusions), and 13 required nasal packing for severe epistaxis. Thrombocytopenia (thrombocyte count ≤ 100 × 10^9^/L) was seen in 614 (95%) of 644 dengue patients. Thrombocyte minimum values (median and 90% range) were similar in the patients with shock (17, 9–46 × 10^9^/L) and in the group with severe bleeding without shock (21, 2–83 × 10^9^/L), and were lower than the minimum values seen in patients without shock or severe bleeding (33, 9–113 × 10^9^/L) (*P* < 0.001, by Mann-Whitney test). Among the 160 patients with confirmed dengue for whom serial coagulation screening tests were performed, the pattern was of significantly increased activated partial thromboplastin times (APTT), reduced fibrinogen levels, and relatively normal prothombin time (PT) measurements similar to the values seen in the OFI group (Table 1).

Abdominal pain and hepatomegaly were seen more commonly in patients with shock than in those without shock (*P* < 0.001, by chi-square test) (Table 1). Visible jaundice was noted in only 11 (< 2%) of 644 dengue patients, of whom 8 had severe bleeding. Acute liver failure developed and markedly deranged coagulation test results were observed for five of these patients, none of whom had shock; one patient died. Jaundice appeared relatively late, commonly during the second week of illness several days after the expected time for shock (median [range]) day of onset (7 days [6–12 days]) and was minor in all cases except those with acute liver failure. The infecting serotype (dengue 2 virus) was identified in only one case with jaundice. Information on ingestion of potentially hepatotoxic medications was limited because most patients did not know what medications they had been prescribed before hospital admission. However, more than 90% of the patients in the study admitted taking standard doses of paracetamol for symptomatic relief, with the pattern of ingestion similar in those with and without severe liver involvement. Although we attempted to gather information on prior alcohol consumption, the response rate was poor and the data collected were believed to be unreliable, precluding analysis.

### Changes in liver function test results.

**Figure 1.**
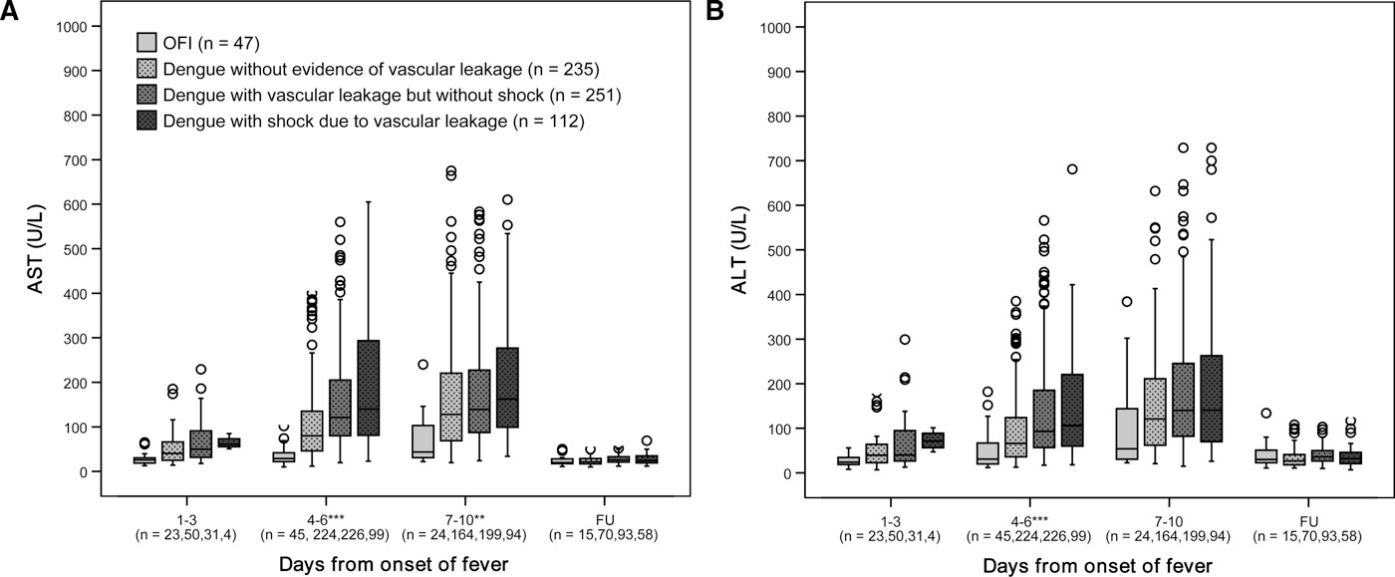
Box and whisker plots showing serial measurements of A, aspartate aminotransferase (AST) and B, alanine aminotransferase (ALT) levels in adults with confirmed dengue infection shown in groups according to the overall severity of vascular leakage and with results for the group with other febrile illnesses (OFI). Total number of patients included in each group is indicated. Forty-six of 644 dengue patients could not be classified for vascular leakage severity because of missing information and are not included. Cuzick test for trend was used to compare transaminase levels in each period across the three vascular leakage severity categories within the dengue patient group, *** *P* < 0.001 and ** *P* < 0.01. Boxes represent the median and interquartile values. Open circles indicate outlying values but extremes are not shown.

Bilirubin levels fell within the normal range for almost all patients except those with visible jaundice. Serial transaminase levels are shown in Figure 1 and Table 2 according to the severity of vascular leakage or bleeding and grouped according to timing during the evolution of the illness. When we considered all dengue patients, only 22 (3%) of 644 had normal aspartate aminotransferase (AST) and alanine aminotransferase (ALT) levels (≤ 40 IU/L) throughout the course of their illness, compared with 23 (49%) of 47 of the OFI patients. In the dengue group, AST and ALT levels began to increase slightly in the early febrile period: median (90% range) of 43 IU/L (18–314 IU/L) for AST levels and 40 IU/L (14–236 IU/L) for ALT levels compared with 24 IU/L (13–68 IU/L) for AST levels and 32 IU/L (13–101 IU/L) for ALT levels at follow-up (*P* < 0.001 for AST and *P* = 0.02 for ALT, by Wilcoxon signed-rank test). Both enzyme levels increased significantly in the critical period to 107 IU/L (30–483 IU/L) for AST and 83 IU/L (22–422 IU/L) for ALT, and reached peak concentrations of 138 IU/L (45–547 IU/L) for AST and 136 IU/L (31–574 IU/L) for ALT during the convalescent period (*P* < 0.001 for comparisons of critical and convalescent values with followup values and *P* < 0.01 for comparisons of critical values with convalescent values, by Wilcoxon signed-rank test).

The highest enzyme levels were recorded in patients with jaundice (median [90% range] of 1,663 IU/L [320–8,680 IU/L] for maximum AST levels during the disease course and 971 IU/L [334–9,917 IU/L] for maximum ALT levels). Levels had returned to the expected normal range at the one month followup visit in 203 (88%) of 231 dengue patients for AST and in 145 (63%) of 231 dengue patients for ALT. The ALT levels were significantly higher than AST levels in OFI patients in the critical and convalescent periods (*P* < 0.05, by Wilcoxon signed-rank test). In contrast, AST levels were consistently higher than ALT levels in the critical and convalescent periods in dengue patients (*P* < 0.01, by Wilcoxon signed-rank test).

In terms of correlation between transaminase levels and markers of disease severity, during the critical period, AST and ALT levels were significantly higher in the dengue patients who experienced shock compared with those without shock (*P* < 0.01, by Mann-Whitney test). This association was still apparent during the convalescent period for AST (*P* < 0.05, by Mann-Whitney test), but ALT levels were similar in the two groups at this time. A similar pattern was observed when we examined associations between transaminase levels and the more detailed grading of vascular leakage severity, excluding the 46 patients who could not be classified due to lack of information (Figure 1). In addition, the AST and ALT levels for dengue patients correlated significantly with bleeding severity in the critical and convalescent periods (*P* < 0.001, by Cuzick test for trend) (Table 2). Both liver enzyme levels demonstrated weak negative correlations with the lowest thrombocyte count and plasma fibrinogen level recorded during the same period (Spearman correlations = –0.2 to –0.3; all *P* < 0.05) and weak positive correlations with the APTT (Spearman correlations = 0.2–0.3; all *P* < 0.05) in the critical and convalescent periods. The PT showed a weak association with transaminase levels only in the convalescent period (Spearman correlation = 0.2; both *P* < 0.05).

### Effect of chronic HBV or HCV co-infection on bleeding severity and laboratory results.

Serologic results for antibodies to HCV were positive in only 3 (2 dengue patients and 1 OFI patient) of the 208 patients tested. Evidence for prior exposure to HBV (antibodies to HBc) was found in 344 (50%) of 694 patients tested, and evidence for chronic HBV infection (antibodies to HBc with positive results for hepatitis B surface antigen and negative results for IgM to HBc) was detected in 69 (11%) of 618 dengue patients and 9 (19%) of 47 OFI patients. Acute HBV infection (IgM to HBc) was not found in any patient. In the dengue group, ALT levels in the critical period were modestly but significantly higher in patients with chronic HBV co-infection than in patients without co-infection (*P* = 0.001, by Mann-Whitney test). A small increase in AST levels was also observed in the critical period but this was not statistically significant. No association was demonstrated between AST or ALT levels and HBV co-infection by the convalescent period (Table 3). In addition, HBV infection had no effect on bleeding severity, vascular leakage severity or development of shock, severity of thrombocytopenia, or on any of the coagulation screening tests in the dengue patients. Infection with HBV was noted in only one of the eleven patients with jaundice and in none of those with acute liver failure.

## Discussion

This prospective observational study presents a detailed description of the evolution of the clinical manifestations of dengue in a large group of adults admitted to an infectious disease hospital in southern Vietnam. Because the Hospital for Tropical Diseases is a referral hospital and recruitment was focused on patients admitted to the AICU, findings generally represent the more severe end of the disease spectrum. However, 546 (74%) of 740 patients enrolled in the study went directly to the hospital. The main finding was that mild-to-moderate hepatic dysfunction, assessed by serial measurements of liver enzyme levels, was apparent in almost all dengue patients and correlated with other measures of severity, although the most abnormal results were noted rather later than during the generally accepted critical period for other complications.

Transaminase levels began to increase from an early stage (day 1–3 of illness) and peaked during the second week of illness. Severe liver involvement (acute liver failure and/or jaundice) was rare, but also commonly occurred during the second week of illness. By follow-up, AST levels had returned to normal levels in most patients, but ALT levels remained slightly increased above the normal range in approximately one-third of the patients. This general pattern, with AST increasing more quickly and peaking at a higher level and then reverting to normal sooner than ALT levels, is unusual and differs from that commonly seen during acute hepatitis caused by hepatitis viruses,^26^ but is similar to observations in studies of dengue in children and adults.^14^–^16^ In addition, the effect of chronic HBV co-infection was primarily apparent on ALT levels.

Although these results indicate that most adults with dengue infection have some degree of hepatic involvement, an additional non-hepatic source of AST could explain the pattern observed. Alanine aminotransferase is primarily associated with hepatocytes, with minimal activity in cardiac and skeletal muscle, and AST is found in erythrocytes, cardiac and skeletal muscle, and kidney and brain tissue, and is often elevated because of damage to those sources and in response to hepatic damage.^26^ Given the prominence of musculoskeletal symptoms among adults with dengue, skeletal muscle injury could contribute to the elevation in AST levels. The plasma half-life of AST is shorter than that of ALT,^26^ but it is possible that the slower improvement in ALT levels simply reflects slower evolution of the hepatic disease than of the musculoskeletal problems. We were unable to investigate specific muscle enzymes or isoforms in this study, but creatine kinase levels are known to be elevated during the acute phase of dengue infection.^27^ In combination with other simple laboratory tests, transaminase levels, particularly AST levels, have been suggested as a potential marker to help differentiate dengue from other viral infections during the early febrile phase.^28^ However, mild-to-moderate increases in transaminase levels, with AST higher than ALT levels, are a non-specific finding that may be observed in a number of other infections such as typhoid fever and malaria^29^,^30^ and in alcohol-related hepatitis.^26^

In agreement with results of smaller studies, we found associations between transaminase levels and increasing severity of vascular leakage and bleeding severity.^14^–^17^ Acute liver failure developed in only a small number of patients, none of whom had severe vascular leakage. In addition, severe bleeding (requiring intervention or involving a vital organ) was seen in 55 (9%) of 644 dengue-infected adults, most of whom did not have shock. This finding is in contrast to our previous pediatric studies at the same hospital, in which severe bleeding was much less frequent and was almost invariably associated with DSS and prolonged shock.^8^,^10^ Although liver involvement might be expected to contribute to derangements in hemostatic parameters, we only found prolonged PT values in the patients with liver failure. In most other patients, PT values fell within the normal range and were similar to the values seen in the OFI group. We also demonstrated only a weak correlation between PT and transaminase levels during the convalescent period, suggesting that liver synthetic function in terms of coagulation factor production was generally well compensated. We did find evidence of the typical dengue-associated coagulopathy of increased APTT and low fibrinogen levels in most patients, but low thrombocyte counts and dysfunction of the remaining thrombocytes are probably the major cause of clinical bleeding. Preliminary comparisons suggest that the thrombocytopenia is more severe in adults than in children, although formal assessment will be necessary to address this question properly.

The pathogenesis of liver involvement during dengue infections is still poorly understood.^31^ Potential mechanisms of hepatic injury involve a variety of potential insults including direct effects of the virus or host immune response on liver cells, circulatory compromise and/or hypoxia caused by hypotension or localized vascular leakage inside the liver capsule, hepatotoxic effects of drugs such as acetaminophen or traditional herbal remedies, and tissue tropism of particular viral serotypes or genotypes. Given the fact that many organ systems are deranged by the time a patient dies, histopathologic findings from autopsy studies are of limited value in attempting to clarify primary disease mechanisms, and unfortunately suitable biopsy material is rarely available from less severe cases. The magnitude and evolution of the liver enzyme changes demonstrated in this study and the relationships that we observed with other markers of disease severity favor an adverse effect of immune dysregulation over a direct viral effect as the likely primary mechanism responsible for the hepatic dysfunction in the most patients. Dengue virus is rapidly cleared from plasma during the first few days of symptoms and viral RNA is rarely detectable after the first week of illness.^32^ A direct viral effect might be expected to be maximal early in the infection coinciding with peak viremia, although it is plausible that virus remains sequestered in the liver for some time after clearance from plasma. However, the host immune response is well established by the time the critical phase (day 4–6 of illness) is reached, particularly in secondary infections. There is a body of evidence supporting the view that complications such as shock and thrombocytopenia are primarily immune mediated, potentially involving a complex interaction between cytokines, activated T cells in tissues, immune complexes, and other immune effector mechanisms.^2^,^33^

Regarding the effects of concomitant infection with other hepatitis viruses common in Asia, we found a significant increase in ALT levels in dengue patients with chronic HBV infection compared with those without infection. However, the effect was minor and did not affect coagulation parameters, bleeding severity, or clinical signs of liver disease. In the past, several small studies have shown no effect of HBV infection on acute dengue morbidity,^14^,^15^ but our findings are in agreement with a recent study in which nearly 400 patients with dengue-1 virus infection were studied and showed slightly greater increases in ALT levels among patients with HBV co-infection.^23^ We found no clinically significant effects in our study, but the possibility that repeated dengue infections may alter the rate of progression of the HBV infection needs to be considered. Few patients with HCV infection were identified and we were unable to assess the effects of co-infection with this virus.

In summary, among nearly 650 adults with dengue recruited over two years at one institution, liver involvement demonstrated by increases in transaminase levels occurred in almost all patients and correlated with disease severity in terms of vascular leakage and bleeding. Jaundice and acute liver failure developed in only a small proportion of patients and occurred relatively late in the disease course, usually without evidence of vascular leakage severe enough to cause shock. Severe bleeding was more frequent, but also occurred commonly in the absence of major vascular leakage. The pathogenesis of liver involvement is still unknown but likely represents an adverse effect of the host immune response that is proportional to the initial viral burden. In most patients, the effect is mild and full recovery is usual with supportive care. In the rare cases in which severe liver disease develops, additional mechanisms are probably involved but given the small number of cases, it is likely to prove difficult to identify the factors contributing to such idiosyncratic responses.

## Figures and Tables

**Table 1:** Demographic, summary clinical information, and coagulation tests results for adults with confirmed dengue infection (with and without shock) and those with other febrile illnesses*

Characteristic	Other febrile illness (n = 47)	Confirmed dengue (n = 644)		
		No shock (n = 532)	Shock (n = 112)	*P*†
Age, years	21 (16–49)	22 (15–35)	20 (15–32)	0.02
Male sex	21 (45)	268 (50)	49 (44)	0.20
Day of illness on enrollment	4 (2–6)	5 (3–7)	6 (4–7)	< 0.001
Any skin bleeding‡	16 (34)	445 (84)	109 (98)	< 0.001
Any mucosal bleeding‡	15 (32)	293 (55)	80 (72)	< 0.001
Bleeding severity‡				
No bleeding	24 (51)	54 (10)	0	–
Skin bleeding only	8 (17)	185 (35)	30 (27)	0.12
Minor mucosal bleeding with or without skin bleeding	15 (32)	253 (48)	66 (60)	0.02
Severe bleeding, any site	0	40 (8)	15 (14)	0.04
Abdominal pain§	20 (43)	303 (58)	93 (85)	< 0.001
Liver palpable¶	5 (11)	141 (27)	83 (74)	< 0.001
Liver enlarged > 2 cm¶#	0	15 (3)	13 (12)	0.27
Jaundice	0	9 (2)	2 (2)	0.94
Acute liver failure**	0	5 (1)	0	–
Hospital stay, days	5 (4–7)	5 (2–8)	5 (2–12)	0.25
Death††	0	1 (< 1)	5 (5)	–
Lowest platelet count × 10^9^/L	103 (36–170)	32 (9–111)	17 (9–46)	< 0.001
Highest PT value,‡‡ seconds	16.6 (14.9–20.5)	16.1 (13.3–20.2)	16.7 (14.5–27)	0.03
Highest APTT value,‡‡ seconds	38.1 (32–56.4)	45.3 (33.9–62.7)	50.5 (38.8–120)	< 0.001
Lowest fibrinogen level,‡‡ g/L	3.8 (2.2–4.9)	2.8 (1.7–3.9)	2.1 (0.5–3.8)	< 0.001

* Values are no. (%) for categorical variables and median (90% range) for continuous variables. PT = prothrombin time; APTT = activated partial thromboplastin time.

† *P* value for comparisons between confirmed dengue infection with and without shock.

‡ Missing data for one patient in confirmed dengue infection group with shock.

§ Missing data for 9 and 2 patients in confirmed dengue infection groups without and with shock, respectively.

¶ Missing data for one patient in confirmed dengue infection without shock.

# Liver palpable > 2 cm below the costal margin in the mid-clavicular line.

** Development of any degree of mental alteration with elevated liver enzyme levels and an international normalized ratio ≥ 1.5 in a patient without evidence of pre-existing cirrhosis.

†† Causes of death: 1 acute liver failure, 1 shock plus encephalopathy, 2 shock plus severe bleeding, 1 prolonged shock, and 1 shock with multi-organ failure.

‡‡ No. patients with results for coagulation screening tests for other febrile illness, no shock, and shock groups are 20, 122, and 38, respectively.

**Table 2:** Transaminase levels according to the severity of bleeding in adults with confirmed dengue infection and in the other febrile illness group*

	AST (U/L)				ALT (U/L)			
	D1–3	D4–6†	D7–10†	FU	D1–3	D4–6†	D7–10†	FU
Other illness group (n = 47) (n = 23, 45, 24, 15)‡	26	29	44	20	24	31	54	30
	13–105	12–247	23–553	11–65	8–127	16–257	24–575	11–102
Confirmed dengue (n = 644)								
No bleeding (n = 15, 50, 31, 9)‡	42	58	108	24	29	46	93	26
	18–141	23–324	30–239	17–33	7–115	17–250	23–270	18–47
Skin bleeding only (n = 35, 205, 154, 70)‡	47	104	123	23	43	78	109	30
	20–316	34–359	47–449	13–58	17–371	25–354	36–448	12–102
Minor mucosal bleeding (n = 41, 289, 255, 124)‡	41	117	151	25	39	98	151	37
	14–185	30–527	46–539	13–78	13–209	23–428	31–553	14–128
Severe bleeding (n = 4, 38, 45, 28) ‡	322	136	184	24	166	140	148	28
	51–1,008	25–2,546	34–4,520	12–72	47–251	26–2,816	20–4,045	12–100

* Values are median and 90% range. AST = aspartate aminotransferase; ALT = alanine aminotransferase; D = day; FU = followup.

† Cuzick test for trend was used to compare transaminase levels across the four bleeding severity categories within the dengue patient group (*P* < 0.001).

‡ No. of patients in early febrile (D1–3), critical (D4–6), convalescent (D7–10) periods and at follow-up, respectively.

**Table 3:** Effect of chronic hepatitis B virus co-infection on bleeding severity and transaminase levels in 618 adults with dengue*

Characteristic	Chronic HBV co-infection (n = 69)	No evidence for HBV infection (n = 549)	*P*†
Severe bleeding	7 (10)	44 (8)	0.55
Mucosal bleeding or any severe bleeding	46 (67)	312 (57)	0.12
AST in critical period‡	122 (28–548)	105 (30–483)	0.07
ALT in critical period‡	118 (29–520)	81 (22–385)	0.001
AST in convalescent period§	152 (58–443)	137 (41–563)	0.68
ALT in convalescent period§	144 (50–637)	135 (30–581)	0.21

* Values are no. (%) for categorical variables and median (90% range) for continuous variables. HBV = hepatitis B virus; AST = aspartate aminotransferase; ALT = alanine aminotransferase.

† Chi-square test or Fisher's exact test or Mann-Whitney test were used as appropriate.

‡ No. of patients with and without chronic HBV co-infection in critical period are 62 and 498, respectively.

§ No. of patients with and without chronic HBV co-infection in convalescent period are 56 and 417, respectively.

## References

[1] Mackenzie JS Gubler DJ Petersen LR 2004 Emerging flaviviruses: the spread and resurgence of Japanese encephalitis, West Nile and dengue viruses Nat Med 10 S98 S109 15577938 10.1038/nm1144

[2] Halstead SB 2007 Dengue Lancet 370 1644 1652 17993365 10.1016/S0140-6736(07)61687-0

[3] World Health Organization 1997 Dengue Haemorrhagic Fever: Diagnosis, Treatment, Prevention and Control Second edition Geneva World Health Organization

[4] World Health Organization 2009 Dengue: Guideline for Diagnosis, Treatment, Prevention and Control Geneva World Health Organization

[5] Harris E Videa E Perez L Sandoval E Tellez Y Perez ML Cuadra R Rocha J Idiaquez W Alonso RE Delgado MA Campo LA Acevedo F Gonzalez A Amador JJ Balmaseda A 2000 Clinical, epidemiologic, and virologic features of dengue in the 1998 epidemic in Nicaragua Am J Trop Med Hyg 63 5 11 11357995 10.4269/ajtmh.2000.63.5

[6] Wichmann O Hongsiriwon S Bowonwatanuwong C Chotivanich K Sukthana Y Pukrittayakamee S 2004 Risk factors and clinical features associated with severe dengue infection in adults and children during the 2001 epidemic in Chonburi, Thailand Trop Med Int Health 9 1022 1029 15361117 10.1111/j.1365-3156.2004.01295.x

[7] Wills BA Nguyen MD Ha TL Dong TH Tran TN Le TT Tran VD Nguyen TH Nguyen VC Stepniewska K White NJ Farrar JJ 2005 Comparison of three fluid solutions for resuscitation in dengue shock syndrome N Engl J Med 353 877 889 16135832 10.1056/NEJMoa044057

[8] Wills BA Oragui EE Stephens AC Daramola OA Dung NM Loan HT Chau NV Chambers M Stepniewska K Farrar JJ Levin M 2002 Coagulation abnormalities in dengue hemorrhagic fever: serial investigations in 167 Vietnamese children with dengue shock syndrome Clin Infect Dis 35 277 285 12115093 10.1086/341410

[9] Carlos CC Oishi K Cinco MT Mapua CA Inoue S Cruz DJ Pancho MA Tanig CZ Matias RR Morita K Natividad FF Igarashi A Nagatake T 2005 Comparison of clinical features and hematologic abnormalities between dengue fever and dengue hemorrhagic fever among children in the Philippines Am J Trop Med Hyg 73 435 440 16103617

[10] Wills B Tran VN Nguyen TH Truong TT Tran TN Nguyen MD Tran VD Nguyen VV Dinh TT Farrar J 2009 Hemostatic changes in Vietnamese children with mild dengue correlate with the severity of vascular leakage rather than bleeding Am J Trop Med Hyg 81 638 644 19815879 10.4269/ajtmh.2009.08-0008

[11] Kittigul L Pitakarnjanakul P Sujirarat D Siripanichgon K 2007 The differences of clinical manifestations and laboratory findings in children and adults with dengue virus infection J Clin Virol 39 76 81 17507286 10.1016/j.jcv.2007.04.006

[12] Ong A Sandar M Chen MI Sin LY 2007 Fatal dengue hemorrhagic fever in adults during a dengue epidemic in Singapore Int J Infect Dis 11 263 267 16899384 10.1016/j.ijid.2006.02.012

[13] Wang CC Lee IK Su MC Lin HI Huang YC Liu SF Wu CC Lin MC 2009 Differences in clinical and laboratory characteristics and disease severity between children and adults with dengue virus infection in Taiwan, 2002 Trans R Soc Trop Med Hyg 103 871 877 19500813 10.1016/j.trstmh.2009.04.024

[14] Kuo CH Tai DI Chang-Chien CS Lan CK Chiou SS Liaw YF 1992 Liver biochemical tests and dengue fever Am J Trop Med Hyg 47 265 270 1355950 10.4269/ajtmh.1992.47.265

[15] Nguyen TL Nguyen TH Tieu NT 1997 The impact of dengue haemorrhagic fever on liver function Res Virol 148 273 277 9272578 10.1016/s0923-2516(97)88364-1

[16] Souza LJ Alves JG Nogueira RM Gicovate Neto C Bastos DA Siqueira EW Souto Filho JT Cezario Tde A Soares CE Carneiro Rda C 2004 Aminotransferase changes and acute hepatitis in patients with dengue fever: analysis of 1,585 cases Braz J Infect Dis 8 156 163 15361994 10.1590/s1413-86702004000200006

[17] Mohan B Patwari AK Anand VK 2000 Hepatic dysfunction in childhood dengue infection J Trop Pediatr 46 40 43 10730040 10.1093/tropej/46.1.40

[18] Bhamarapravati N 1989 Hemostatic defects in dengue hemorrhagic fever Rev Infect Dis 11 (Suppl 4) S826 S829 2665014 10.1093/clinids/11.supplement_4.s826

[19] Huerre MR Lan NT Marianneau P Hue NB Khun H Hung NT Khen NT Drouet MT Huong VT Ha DQ Buisson Y Deubel V 2001 Liver histopathology and biological correlates in five cases of fatal dengue fever in Vietnamese children Virchows Arch 438 107 115 11253111 10.1007/s004280000329

[20] Rosen L Khin MM U T 1989 Recovery of virus from the liver of children with fatal dengue: reflections on the pathogenesis of the disease and its possible analogy with that of yellow fever Res Virol 140 351 360 2772416 10.1016/s0923-2516(89)80115-3

[21] Rosen L Drouet MT Deubel V 1999 Detection of dengue virus RNA by reverse transcription-polymerase chain reaction in the liver and lymphoid organs but not in the brain in fatal human infection Am J Trop Med Hyg 61 720 724 10586901 10.4269/ajtmh.1999.61.720

[22] Jessie K Fong MY Devi S Lam SK Wong KT 2004 Localization of dengue virus in naturally infected human tissues, by immunohistochemistry and *in situ* hybridization J Infect Dis 189 1411 1418 15073678 10.1086/383043

[23] Tang Y Kou Z Tang X Zhang F Yao X Liu S Jin X 2008 Unique impacts of HBV co-infection on clinical and laboratory findings in a recent dengue outbreak in China Am J Trop Med Hyg 79 154 158 18689615

[24] Polson J Lee WM 2005 AASLD position paper: the management of acute liver failure Hepatology 41 1179 1197 15841455 10.1002/hep.20703

[25] Hang VT Nguyet NM Trung DT Tricou V Yoksan S Dung NM Van Ngoc T Hien TT Farrar J Wills B Simmons CP 2009 Diagnostic accuracy of NS1 ELISA and lateral flow rapid tests for dengue sensitivity, specificity and relationship to viremia and antibody responses PLoS Negl Trop Dis 3 e360 19156192 10.1371/journal.pntd.0000360 PMC2614471

[26] Rigato I Ostrow JD Tiribelli C 2007 Biochemical investigations in the management of liver disease Rodes J Textbook of Hepatology: From Basic Science to Clinical Practice Third edition Boston, MA Blackwell Publishing 451 467

[27] Villar-Centeno LA Diaz-Quijano FA Martinez-Vega RA 2008 Biochemical alterations as markers of dengue hemorrhagic fever Am J Trop Med Hyg 78 370 374 18337328

[28] Kalayanarooj S Vaughn DW Nimmannitya S Green S Suntayakorn S Kunentrasai N Viramitrachai W Ratanachu-eke S Kiatpolpoj S Innis BL Rothman AL Nisalak A Ennis FA 1997 Early clinical and laboratory indicators of acute dengue illness J Infect Dis 176 313 321 9237695 10.1086/514047

[29] Patwari A Aneja S Berry AM Ghosh S 1979 Hepatic dysfunction in childhood malaria Arch Dis Child 54 139 141 373643 10.1136/adc.54.2.139 PMC1545372

[30] Morgenstern R Hayes PC 1991 The liver in typhoid fever: always affected, not just a complication Am J Gastroenterol 86 1235 1239 1882803

[31] Seneviratne SL Malavige GN de Silva HJ 2006 Pathogenesis of liver involvement during dengue viral infections Trans R Soc Trop Med Hyg 100 608 614 16483623 10.1016/j.trstmh.2005.10.007

[32] Vaughn DW Green S Kalayanarooj S Innis BL Nimmannitya S Suntayakorn S Endy TP Raengsakulrach B Rothman AL Ennis FA Nisalak A 2000 Dengue viremia titer, antibody response pattern, and virus serotype correlate with disease severity J Infect Dis 181 2 9 10608744 10.1086/315215

[33] Mathew A Rothman AL 2008 Understanding the contribution of cellular immunity to dengue disease pathogenesis Immunol Rev 225 300 313 18837790 10.1111/j.1600-065X.2008.00678.x

